# B-cell subpopulations in humans and their differential susceptibility to depletion with anti-CD20 monoclonal antibodies

**DOI:** 10.1186/ar3908

**Published:** 2013-03-25

**Authors:** Maria J Leandro

**Affiliations:** 1 Centre for Rheumatology and Bloomsbury Rheumatology Unit, Rayne Building, Room 416, University College London, 5 University Street, London WC1E 6JF, UK

## Abstract

In humans, different B-cell subpopulations can be distinguished in peripheral blood and other tissues on the basis of differential expression of various surface markers. These different subsets correspond to different stages of maturation, activation and differentiation. B-cell depletion therapy based on rituximab, an anti-CD20 mAb, is widely used in the treatment of various malignant and autoimmune diseases. Rituximab induces a very significant depletion of B-cell subpopulations in the peripheral blood usually for a period of 6 to 9 months after one cycle of therapy. Cells detected circulating during depletion are mainly CD20 negative plasmablasts. Data on depletion of CD20-expressing B cells in solid tissues are limited but show that depletion is significant but not complete, with bone marrow and spleen being more easily depleted than lymph nodes. Factors influencing depletion are thought to include not only the total drug dose administered and distribution into various tissues, but also B-cell intrinsic and microenvironment factors influencing recruitment of effector mechanisms and antigen and effector modulation. Available studies show that the degree of depletion varies between individuals, even if treated with the same dose, but that it tends to be consistent in the same individual. This suggests that individual factors are important in determining the final extent of depletion.

## Introduction to B-cell subpopulations

**Figure 1:**
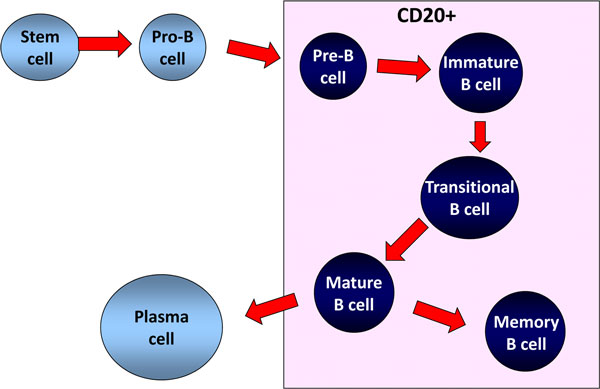
**Simplified scheme of B-cell subpopulations in humans and CD20 expression**.

In humans from birth all new B cells originate from common precursors in the bone marrow. In the bone marrow, peripheral blood and secondary lymphoid tissues, different B-cell subpopulations can be distinguished corresponding to different stages of maturation, activation and differentiation. B-cell subpopulations are characterised mainly by the differential expression of different cell surface markers that include various cluster of differentiation (CD) molecules and different surface immunoglobulin isotypes (B-cell antigen receptor). B-cell development can be separated into an earlier antigen-independent phase, which takes place in the bone marrow, and a later antigen-dependent phase that takes place mainly in secondary lymphoid tissues. In a simplified way, the different B-cell lineage subsets include pro-B cells, pre-B cells, immature and transitional B cells, mature naïve B cells, memory B cells, plasmablasts and plasma cells (Figure 1). Plasmablasts are recently differentiated antibody-producing cells that are usually short-lived but can recirculate and home to tissues such as the mucosa or the bone marrow, where they can differentiate into fully mature plasma cells. In addition, centroblasts and centrocytes are B cells participating in germinal centre reactions.

B-cell precursor subpopulations are found in the bone marrow. In the peripheral blood, transitional, naïve mature and memory B cells and plasmablasts, and more rarely plasma cells, can be identified. Plasma cells are more frequently seen in the bone marrow and peripheral lymphoid tissues. Centrocytes and centroblasts are found in secondary lymphoid tissues where germinal centre reactions take place, and are not found circulating in peripheral blood. Marginal zone B cells can be found in the marginal zone of the spleen and similar populations are described in particular locations in other secondary lymphoid tissues [1]. Marginal zone B cells in human adults are mainly memory B cells. There is still controversy on what drives formation of human marginal zone B cells, to what extent they are similar to mice marginal zone B cells and what is their relationship with circulating IgM^+ ^memory B-cell subsets [1,2].

Immunophenotyping of B cells with multiparameter flow cytometry has allowed identification of an increasing number of different subpopulations, increasing our knowledge of normal B-cell biology and, in particular, changes associated with different disease states. For example, different memory B-cell subsets have now been described in peripheral blood including subsets that do not express CD27, a marker previously thought to be present on all memory B cells [3,4]. Memory B-cell subpopulations include pre-switch IgD^+^IgM^+^CD27^+ ^memory B cells, IgD^-^IgM^+^CD27^+ ^memory B cells (IgMonly memory B cells), post-switch IgA^+^CD27^+ ^and IgG^+^CD27^+ ^memory B cells and also IgA^+^CD27^- ^and IgG^+^CD27^- ^memory B cells [5]. These memory subpopulations show different frequencies of somatic mutation and different replication histories that are thought to reflect their formation on primary or secondary germinal centres or outside germinal centre reactions [5]. A potential new marker for human memory B-cell subpopulations has been identified recently [6]. A proposal has been made that immunophenotyping of peripheral blood B cells should include the markers CD19, CD20, CD24, CD27, CD38 and IgD to be able to distinguish the major subpopulations [7]. More detailed information including separation into further subsets and subtle differences in activation status that may be important when looking at disease states may require use of other markers such as different immunoglobulin isotopes, activation markers or chemokine receptors [6,8-14].

## Anti-CD20 monoclonal antibodies-rituximab

Anti-CD20 mAbs were developed in the late 1980s and in the 1990s for the treatment of non-Hodgkin's lymphoma of B-cell origin. Rituximab (MabThera^®^, Rituxan^®^; Roche, Basel, Switzerland) was licensed for the treatment of follicular lymphoma in 1997/98 and later for diffuse large non-Hodgkin's lymphoma and chronic lymphocytic leukaemia. In 2006 rituximab was licensed for the treatment of rheumatoid arthritis (RA). Rituximab is also used off-license for the treatment of other B-cell malignant diseases, in transplantation and for the treatment of a variety of other autoimmune diseases, predominantly diseases associated with the presence of autoantibodies. Various other therapeutic anti-CD20 mAbs are either available on the market (Ofatumumab-Arzerra^®^; GlaxoSmithKlein, UK-licensed for the treatment of chronic lymphocytic leukaemia), undergoing clinical trials or under development [15].

The CD20 antigen is expressed by the majority of cells in the B-lymphocyte lineage, but not by haematopoietic stem cells, the earliest B-cell precursors (pro-B cells) or terminally differentiated plasmablasts and plasma cells (Figure 1). The CD20 molecule is a transmembrane protein thought to function as a calcium channel and to be involved in B-cell activation and proliferation. A recent case report of a patient with CD20 deficiency suggested a role in T-cell-independent antibody responses[16].

Because haematopoietic stem cells are not directly depleted by anti-CD20 antibodies, one course of treatment with rituximab is followed by B-cell repopulation of the peripheral blood starting usually within 6 to 9 months-but it can take several months or even years for total B-cell numbers in the peripheral blood to recover to pretreatment levels. Repopulation occurs mainly with naïve B cells, with increased frequency and numbers of transitional B cells similar to that seen after bone marrow transplantation [14,17]. The time at which B-cell repopulation of the peripheral blood starts is probably determined by the extent of earlier depletion, drug clearance and the capacity of the bone marrow to regenerate. Variability in time to repopulation in primate animal models did not seem to be dose dependent [18]. Factors influencing B-cell precursor formation in humans are poorly understood, as are factors that determine to what extent a fully functional B-cell repertoire is regenerated and how long it takes. Whether age or other individual characteristics influence repopulation is not known [19,20].

The fact that plasma cells are also not directly depleted by anti-CD20 antibodies explains why, in the majority of patients, serum total immunoglobulin levels remain within the normal range after treatment with one course of rituximab. Several studies have shown that serum levels of several autoantibodies decrease after treatment with rituximab (although they do not usually become undetectable) and do so proportionally more than total immunoglobulin levels or anti-microbial antibodies [21-23]. This observation suggests that these auto-antibodies are produced by proportionally more short-lived plasma cells and therefore are more dependent on the formation of new plasma cells, which is interrupted by B-cell depletion [23].

Treatment with rituximab is associated with major depletion of normal B cells *in vivo*. Depletion in the peripheral blood is frequently higher than 99% but depletion in other tissues has been less well studied, with several studies documenting that depletion in solid tissues with rituximab is frequently not complete and can show considerable variation between individuals. *In vitro*, rituximab depletes malignant B cells by antibody-dependent cellular cytotoxicity, complement-mediated cytotoxicity and induction of apoptosis. *In vivo*, rituximab is thought to act mainly by inducing antibody-dependent cellular cytotoxicity with activation of complement also contributing [24]. One of the consistent findings in several of the animal and earlier human studies is the variability of depletion seen with anti-CD20 mAbs in different individuals even when treated with the same dose [18,25,26]. Interestingly, depletion in the same individual tends to be consistent in different tissues, suggesting that individual characteristics are important.

## Resistance to depletion with anti-CD20 monoclonal antibodies

Because depletion is achieved by binding of the mAbs to the cell surface CD20 molecules, the final extent of depletion will necessarily depend on the relationship between total number of B cells and total dose of rituximab administered, on accessibility of the drug and effector immune cells to the tissues where B cells are located, on intrinsic or extrinsic factors that may influence B-cell survival and on the efficacy of recruited host immune mechanisms responsible for depletion.

Former small dose-ranging studies in lymphoma and in animal models have shown that B cells in the peripheral blood are readily killed by anti-CD20 antibodies but that higher doses and higher serum levels are needed for depletion in extravascular sites [18,24,25].

Factors influencing antigen and effector modulation are thought to be important in determining the final extent of depletion achieved (Table 1) [18,27,28]. Antigen modulation refers to antigen endocytosis/modulation after binding to the antibody. Contrary to what was originally thought, this can be seen with the CD20 molecule after binding with certain anti-CD20 antibodies including rituximab [29]. This can lead to less recruitment of Fcγ receptors on effector immune cells and to decreased serum drug levels. Effector modulation refers to genetic and acquired mechanisms that can enhance or diminish effector immune cell function and therefore influence the extent of depletion. For example, a Fcγ receptor IIIa polymorphism that can influence affinity for IgG has been associated with clinical response in lymphoma [28]. Profound complement depletion as seen during treatment of chronic lymphocytic leukaemia with rituximab can be a limiting factor for further depletion [28].

**Table 1:** Potential mechanisms of resistance or of susceptibility to depletion by anti-CD20 monoclonal antibodies

Depletion	Mechanisms
B-cell and antigen related	Lack of CD20 surface expression
	CD20 (antigen) modulation/endocytosis
	Lipid raft composition
	Expression of complement regulatory proteins
Immune host phenomena related	FcγRIIIA polymorphisms
	FcγRIIB expression
	C1q polymorphisms
	Exhaustion of cytotoxic mechanisms (for example, complement)

Mechanisms reviewed in [24,28].

Intrinsic B-cell factors that may influence depletion include high expression of complement regulatory proteins as seen in chronic lymphocytic leukaemia [28]. In cynomolgus monkeys, different sensitivities to rituximab were associated with, but not fully explained by, different levels of expression of CD20 [30]. Binding of rituximab to CD20 leads to translocation of the CD20 molecule to lipid rafts. Alterations in lipid raft composition and treatment with statins have been associated with less good responses to rituximab [28]. To what extent external B-cell survival factors, in particular the cytokine B-cell activating factor (BAFF), influence depletion is not known, although it has been suggested that local high levels of BAFF may contribute to resistance to depletion by rituximab [31].

In animal models, certain subpopulations have been shown to be more resistant to depletion with anti-CD20 antibodies but this varies with the mice strain used and whether they were studies using human CD20 transgenic mice treated with anti-human CD20 mAbs or non-transgenic mice treated with anti-mouse CD20 mAbs [32,33]. Populations that were found to be more resistant to depletion were peritoneal B1-type B cells, germinal centre B cells and marginal zone B cells [32,33]. Insufficient depletion of peritoneal B1 cells is thought to be due to the lack of effector cells in the peritoneal space [33]. Differential sensitivity of germinal centre and marginal zone B cells to anti-CD20 antibodies has also been described in cynomologous monkeys, with differences appearing more prominent in the lymph nodes than in the spleen [30]. The relative resistance of some populations is thought to be related to B-cell and micro-environment differences responsible for antigen or effector modulation or related to direct resistance of the B cells involved. In an autoimmune mouse model of lupus, B cells were more resistant to depletion when compared with nonautoimmune mice and more frequent administration of larger doses increased efficacy of depletion [34]. Less good depletion has also been associated with acquired defects in antibody-dependent cellular cytotoxicity in the same autoimmune mouse model of lupus [35].

To what extent the differential susceptibility of various B-cell subsets demonstrated in some of the animal models reflects what happens in humans *in vivo *is not known. Different B-cell malignancies deriving from B cells at different stages of differentiation and different tumour locations are also associated with differential responses to treatment with anti-CD20 mAbs but susceptibility of the correspondent normal human B-cell sub-populations is expected to be substantially different. Whether there are any differences in susceptibility to depletion of autoreactive human B-cell clones when compared with nonautoreactive ones, as suggested by mouse models [34], and whether there are any significant differences in susceptibility to depletion of disease-associated B-cell clones between different autoimmune diseases are also not known.

In addition, administration of chimaeric anti-CD20 mAbs such as rituximab can be associated with formation of human anti-chimaeric antibodies that can influence drug action and clearance. Although most large studies show no association between the presence of human anti-chimaeric antibodies and clinical response or depletion, this association has been described, for example, in small studies in systemic lupus erythematosus patients [36,37].

With evidence showing that not all B cells that bind rituximab are depleted there is an interest in knowing what exactly happens to these cells *in vivo *during the period of depletion. Are they eventually depleted later on, particularly if they recirculate in peripheral blood? Are they functionally impaired? Are they able to expand in an environment with less competition and raised BAFF levels? Kamburova and colleagues tried to address some of these issues by studying the *in vitro *effects of incubation with rituximab on proliferation, activation and differentiation of nondepleted human normal peripheral blood B cells [38]. They reported that incubation with rituximab (for 30 minutes at 5 μl/ml) inhibited the proliferation of stimulated CD27^- ^naïve B cells but not of CD27^+ ^memory B cells and this was associated with a relative increase of B cells with an activated naïve phenotype. B cells stimulated in the presence of rituximab induced stronger T-cell proliferation and the T-cell population showed a more Th2-like phenotype. These results suggest that B cells which are exposed to rituximab but are not depleted may have altered function and that naïve and memory B cell populations may be differentially affected. Whether any of these phenomena occur *in vivo *and what their implications would be are unclear. Interestingly, and similar to what happens after bone marrow transplantation, the residual B cells are not able to expand and repopulate the peripheral blood, even in the presence of abundant BAFF.

### B-cell depletion in peripheral blood

Administration of rituximab is usually associated with a rapid and profound depletion of circulating B cells in the peripheral blood [18]. Major depletion effector cells are probably macrophages from the reticulo-endothelial system [24]. Studies in autoimmune diseases-in particular, RA and systemic lupus erythematosus-have documented variable degrees and durations of B-cell depletion in peripheral blood in different individuals following treatment with rituximab with standard doses [17,36,37,39-41]. Incomplete B-cell depletion in the peripheral blood, as defined by B-cell counts >5 cells/μl after treatment with rituximab, has been well documented in cases of patients with autoimmune diseases, more frequently in systemic lupus erythematosus than in RA [17,36,37]. Persistent presence of circulating B cells has also been documented with high-sensitivity flow cytometry and has been associated with no or less good response to treatment [39,40]. Insufficient depletion can be seen on retreatment with documented very rapid clearance of rituximab in association with a marked human anti-chimaeric antibody response [42]. Other mechanisms underlying incomplete depletion in the peripheral blood have not been well studied but are probably a consequence of more rapid clearance of the drug and/or antigen and effector modulation phenomena [17,24,36,37].

The very small numbers of circulating B cells that can be detected during periods of depletion usually show a phenotype of plasmablasts but cells with memory or even naïve B cells have also been reported [17,40,41,43]. The CD20 antigen cannot usually be detected in these memory B cells, suggesting that it is masked by binding to rituximab because the drug can be detected in the circulation for several months [26]. Mei and colleagues described that, similarly to their controls, the majority of circulating plasmablasts/plasma cells detected during depletion were positive for IgA and a reasonable proportion expressed markers suggesting they had been formed in mucosal tissue and were circulating back to mucosal areas [44]. These results suggest that depletion in mucosal-associated lymphoid tissue may be particularly less pronounced.

Repopulation of the peripheral blood after treatment with a standard dose of rituximab usually starts 6 to 9 months after treatment with predominantly transitional and naïve B cells as previously mentioned. Frequently, repopulation with larger numbers of memory B cells and/or plasmablasts has been associated with earlier relapse [17,40,45]. At repopulation, the decrease from baseline in the frequency of pre-switch memory B cells (CD27^+^IgD^+^)was larger than the decrease in the switched memory B-cell population (CD27^+^IgD^-^) [46]. However, to what extent circulating memory B cells at repopulation are old memory B cells that have not been depleted by rituximab or recently differentiated memory B cells is not known. We therefore do not know whether relative frequencies of the different B-cell subpopulations at repopulation can tell us anything about the subpopulations of cells that may have resisted depletion.

In RA, nonresponse has been associated with higher numbers of plasmablasts before treatment and early relapse has been associated with higher numbers of CD27^+ ^memory B cells before treatment [39,45]. Again, to what extent this may indicate less susceptibility and insufficient depletion of memory B-cell subsets in association with no response or with a shorter response is not known.

### B-cell depletion in bone marrow and secondary lymphoid tissues

Unfortunately, there are limited data on the degree of depletion of normal B cells in secondary lymphoid organs and other solid tissues in human individuals treated with rituximab, and hardly any data on differential susceptibility to depletion of different subpopulations in different tissues except for the expected resistance of CD20^- ^plasmablasts and plasma cells to depletion [47]. Animal studies in primates showed that increasingly higher doses are needed to deplete bone marrow, spleen and lymph nodes in this order [18,48,49]. These studies also showed that B-cell depletion in solid tissues was frequently significant, but not complete, and that it varied from site to site and from individual to individual even when the same doses were used. Interestingly, consistency regarding the degree of depletion achieved in different lymph nodes in the same individual was described [18,20,48,49]. As previously mentioned, mice studies suggested that B cells resident in tissues other than peripheral blood may be partly resistant to depletion by anti-CD20 antibodies either because of local defective effector mechanisms or because the B cells have a particular phenotype that renders them resistant to depletion in association with their specific state of maturation, activation or differentiation.

In bone marrow samples of RA patients treated with rituximab a relatively high number of B-cell precursors subpopulations can be seen [50-52]. This has been documented at 1 month or 3 to 4 months after treatment, at a time when peripheral blood repopulation had not yet started [50,51]. Persistence of CD20^- ^plasma cells has been observed as expected [50,51]. In the two studies where phenotyping was more detailed, the cells found were mainly B-cell precursors and recirculating memory B cells [50,52]. Once again, variability between individuals was observed [50,52].

The presence of cells of B-cell lineage that presumably should be expressing CD20 has therefore been well documented and rituximab is probably still present and binds to the CD20 molecule, preventing its detection in flow cytometry as discussed above [50,51]. Alternatively, antigen endocytosis/modulation could occur. Whether the developing B cells are eventually depleted by anti-CD20 recruited mechanisms or whether their full maturation is prevented by binding of rituximab to CD20 is not known.

In a study of autopsy samples of lymph node and spleen of patients with lymphoma treated with rituximab monotherapy or with rituximab and chemotherapy, a substantial reduction of B-cell populations was documented-with only three out of eight patients showing any reactivity for markers of cells of B-cell lineage in the lymph nodes and only one out of eight in the spleen by immunohistochemistry [53]. Similarly, a study in patients with idiopathic thrombocytopenic purpura showed major and prolonged depletion of B cells in the spleen of 10 patients treated with rituximab [54]. The number of residual B cells correlated with time from rituximab treatment but was <5% of spleen lymphocytes in eight out of nine patients studied up to 10 months after rituximab treatment. Plasma cells were detected at increased frequencies when compared with patients with idiopathic thrombocytopenic purpura not treated with rituximab. In a patient with idiopathic thrombocytopenic purpura, analysis of spleen and bone marrow samples by flow cytometry revealed complete depletion of B cells 3 months after treatment with rituximab [55]. In another patient with idiopathic thrombocytopenic purpura, B cells in the spleen 3 months after rituximab treatment were only present in very low numbers (around 0.1%) [56]. Interestingly, in this later study persistence of memory B cells against vaccinia virus in the spleen of patients previously treated with rituximab was documented [56]. In kidney transplant patients that had as plenectomy 3 to 12 days after treatment with rituximab, naïve B cells were reduced but not memory B cells or plasma cells [57].

Vaccination studies in patients treated with rituximab can provide indirect data on B-cell subpopulations that may be resistant to depletion with anti-CD20 mAbs. However, published data are difficult to interpret because of the small number of patients, effects of concomitant therapy and the background disease itself on the humoral response to vaccines and, in particular, because studies included patients at various stages of B-cell depletion or repopulation at the time of vaccination. Most studies have looked at responses to influenza vaccines and showed absent or decreased humoral responses to vaccination in patients previously treated with rituximab when compared with normal controls or patients not treated with rituximab [58-64]. Some studies described a positive relationship between the antibody responses to vaccination and number of circulating B cells at the time of vaccination [64] or the time from last rituximab treatment [60,62]. Interestingly, when circulating influenza-specific B cells were studied 6 days after vaccination, specific IgM-B cells were decreased in patients treated with rituximab 6 months previously when compared with controls but IgA B cells and IgG B cells were similar [61]. In a study in lymphoma patients, responses to recall antigens in the influenza vaccine were also seen but not to the new antigen [65]. These studies suggest that memory B cells are more resistant to depletion than naïve B cells and can survive treatment with rituximab and be recruited in a secondary immune response.

### B-cell depletion in other solid tissues

In patients with RA, several studies have documented significant but variable depletion of B cells in samples of synovial tissue of involved joints and persistence of CD20^- ^plasma cells [66-68]. Variability in depletion between individuals was not explained by differences in rituximab serum levels [69]. In a study in patients with Sjogren's syndrome, repeated salivary gland biopsies 3 months after treatment with rituximab showed incomplete depletion of B cells [70]. A previous study had shown complete depletion at 4 months [71]. In a study of renal explanted grafts in two patients treated with one dose (4 months earlier) or two doses (10 months earlier) of rituximab, despite depletion of peripheral blood, tertiary lymphoid structures containing B cells were seen [72].

## Conclusion

In summary, although there are several studies looking at the degree and duration of B-cell depletion induced by rituximab in the peripheral blood, there is very little information on the exact degree of depletion in solid tissues - and, in particular, few definite data on whether different subtypes of CD20-expressing B cells are more or less susceptible to depletion by anti-CD20 antibodies. The data available suggest that there is variability between individuals on the extent and duration of depletion induced and that this may have clinical correlations with response and duration of response in autoimmune diseases. Understanding what underlies this variability - and, in particular, whether drug clearance and antigen and effector modulation phenomena are involved - has the potential to lead to more effective B-cell depleting strategies and to increasing our understanding of the role that different B-cell subtypes play in the pathogenesis of the different autoimmune diseases.

## Abbreviations

BAFF: B-cell activating factor; CD: cluster of differentiation; mAb: monoclonal antibody; RA: rheumatoid arthritis; Th: T-helper type.

## Competing interests

The author has received consultancy fees and funding to attend international medical meetings from Roche Pharmaceuticals and consultancy fees and research funding from GlaxoSmithKlein.

## Declarations

This article has been published as part of *Arthritis Research & Therapy *Volume 15 Supplement 1, 2013: B cells in autoimmune diseases: Part 2. The supplement was proposed by the journal and content was developed in consultation with the Editors-in-Chief. Articles have been independently prepared by the authors and have undergone the journal's standard peer review process. Publication of the supplement was supported by Medimmune.
